# A CRISPR-based approach using dead Cas9-sgRNA to detect SARS-CoV-2

**DOI:** 10.3389/fmolb.2023.1201347

**Published:** 2023-06-14

**Authors:** Mustapha Aouida, Maryam Saifaldeen, Dana E. Al-Ansari, Sara Taleb, Ali Ait Hssain, Dindial Ramotar

**Affiliations:** ^1^ College of Health and Life Sciences, Division of Biological and Biomedical Sciences, Hamad Bin Khalifa University, Education City, Qatar Foundation, Doha, Qatar; ^2^ College of Health and Life Sciences, Division of Genomic and Precise Medicine, Hamad Bin Khalifa University, Education City, Qatar Foundation, Doha, Qatar; ^3^ National Heart and Lung Institute, Imperial College London, London, United Kingdom; ^4^ Medical ICU, Department of Medicine, Hamad Medical Corporation, Doha, Qatar; ^5^ Department of Medicine, Weill Cornell Medical College, Doha, Qatar

**Keywords:** SARS-CoV-2, M gene, dead Cas9, restriction enzymes, RT-RPA, diagnostics

## Abstract

Rapid, highly specific, and robust diagnostic kits to detect viruses and pathogens are needed to control disease spread and transmission globally. Of the many different methods proposed to diagnose COVID-19 infection, CRISPR-based detection of nucleic acids tests are among the most prominent. Here, we describe a new way of using CRISPR/Cas systems as a rapid and highly specific tool to detect the SARS-CoV-2 virus using the *in vitro* dCas9-sgRNA-based technique. As a proof of concept, we used a synthetic DNA of the M gene, one of the SARS-CoV-2 virus genes, and demonstrated that we can specifically inactivate unique restriction enzyme sites on this gene using CRISPR/Cas multiplexing of dCas9-sgRNA-*BbsI* and dCas9-sgRNA-*XbaI*. These complexes recognize and bind to the target sequence spanning the *BbsI* and *XbaI* restriction enzyme sites, respectively, and protect the M gene from digestion by *BbsI* and/or *XbaI*. We further demonstrated that this approach can be used to detect the M gene when expressed in human cells and from individuals infected with SARS-CoV-2. We refer to this approach as dead Cas9 Protects Restriction Enzyme Sites, and believe that it has the potential to be applied as a diagnostic tool for many DNA/RNA pathogens.

## Introduction

One of the many challenges in containing the spread of severe acute respiratory syndrome coronavirus 2 (SARS-CoV-2) is the ability to develop a rapid and accurate tool to detect the virus whilst eliminating both false negative and false positive cases. Several diagnostic tests are currently being used, such as the real-time reverse transcriptase-polymerase chain reaction (RT-qPCR) and the Clustered Regularly Interspaced Short Palindromic Repeats (CRISPR)-based approaches detecting the nucleic acids of the virus (Ali et al., 2020; Broughton et al., 2020; Carter et al., 2020; Ding et al., 2020; Xiang et al., 2020; Zhang et al., 2020; Azhar et al., 2021; Rahimi et al., 2021; Ali et al., 2022; Hernandez-Garcia et al., 2022; Ma et al., 2023; Xiao et al., 2023). The RT-qPCR is the gold standard diagnostic test to detect the SARS-CoV-2 virus (Carter et al., 2020). However, it has some limitations as it i) requires centralized, highly sophisticated, and expensive equipment, ii) a skilled technical staff, iii) there are limits in testing capacity, and iv) the timeline of each test (∼2 h) (Tahamtan and Ardebili, 2020).

Diagnostic methods based on CRISPR and CRISPR-associated proteins are among the most promising techniques for the detection of SARS-CoV-2 and its variants because they are accurate, specific, sensitive, simple, rapid, equipment-free, and provide deliverables to the end-users (Ali et al., 2020; Broughton et al., 2020; Carter et al., 2020; Ding et al., 2020; Xiang et al., 2020; Zhang et al., 2020; Azhar et al., 2021; Rahimi et al., 2021; Ali et al., 2022; Hernandez-Garcia et al., 2022; Ma et al., 2023; Xiao et al., 2023). However, these diagnostic tests can sometimes lead to false positives and false negatives due to erroneous binding of the primers that guide RNA onto the target nucleic acid or insufficient viral replication at the early stages of infection to be detected by antigen testing. Thus, there is a need for more accurate approaches to detect the virus.

We have previously demonstrated the ability of the inactive dead Cas9-sgRNA ribonucleoprotein complex (dRNPC) in sheltering restriction enzyme (RE) recognition/cut site, inhibiting DNA cleavage, and applying this approach in DNA cloning (Saifaldeen et al., 2021). We proposed the possibility of using dRNPC to be used as a diagnostic tool for any viral pathogen, given that their nucleic acid sequence is known. Compared to existing CRISPR-based diagnostics, this approach has an additional layer of specificity determined by the blocking activity of the specific restriction enzyme targeted by the dRNPC.

Herein, we developed an *in vitro* approach that uses dRNPCs for the detection of a specific and essential SARS-CoV-2 gene, the M gene, which encodes for the membrane glycoprotein of the virus. The M gene is conserved in the virion and, thus, is a target for many vaccines and rapid antigen tests for diagnosis of viral infectivity (Dolan et al., 2022). In our approach, we also used the M gene since it contains two sites of commonly available REs: *BbsI* and *XbaI*. We followed the published protocol (Saifaldeen et al., 2021) and proved that dRNPC targeting the restriction enzyme site protected the DNA from being cut by the respective restriction enzyme. In addition, we validate the detection of the M gene expressed in HEK293T cells as well as from COVID-19-positive individuals. The advantage of this novel approach is that i) it is rapid, and only 60 min are required to detect the viral M gene, ii) it is cost effective, iii) it is highly accurate, and iv) it will positively identify individuals who are carriers of the SARS-CoV-2. We are referring to this approach as ‘dCPRES,’ short for ‘dead Cas9 Protects Restriction Enzyme Site,’ and we anticipate that it will eliminate both false negatives and false positives generated by the currently used RT-qPCR technique.

## Materials and methods

### Enzymes and reagents

All CRISPR reagents (dCas9, crRNA, and tracrRNA) and primers were purchased from Integrated DNA Technologies (Coralville, IA, United States). All restriction enzymes (*BbsI*, *XbaI*, *BamHI*-HF (high fidelity), and *EcoRI*-HF) and their specific buffers (1.1, 2.1, 3.1, and cut-smarter) were purchased from New England Biolabs (Ipswich, MA, United States). T4 DNA ligase 1 U/µL (Cat. No. 15224025) was purchased from Thermo Fisher Scientific (United States). The DNA sequencing kit BigDyeTM Terminator v3.1 Cycle Sequencing Kit (Cat. No. 4337455) was purchased from Applied-Biosystems.

### Plasmids

The M gene was synthesized by IDT (Coralville, IA, United States) and supplied as a plasmid (pIDTSMARTAmp-M), which was used as a DNA template to amplify the M gene. The pcDNA3.1 (common laboratory stock vector) was used to produce the plasmid pcDNA3.1-CMV-M for expression in mammalian cells.

### Ribonucleoprotein complex formation

The dCas9-sgRNA ribonucleoprotein complex (dRNPC) was prepared as previously described by Saifaldeen et al. (2020) (Saifaldeen et al., 2021). In brief, the sgRNAs targeting *BbsI* and *XbaI* were prepared using two synthetic RNA oligonucleotides, a crRNA, and a tracrRNA, that must be annealed prior to mixing with dCas9 protein. The optimized lengths of crRNA and tracrRNA are 36 and 67 bases, respectively (Saifaldeen et al., 2021). To form the sgRNA, 1 µL of Alt-R crRNAs (100 µM) (Table 1) was combined with 1 µL Alt-R tracrRNA (100 µM) in a 1:1 ratio in 98 µL of Nuclease Free Water IDT (Cat. No. 11-04-02-01) heated to 95°C for 5 min, followed by a steady cool down to 25°C (approx. 1 h). The RNP complex can be stored for up to 4 weeks at 4°C or for up to 6 months at −80°C. The dCas9 and sgRNA were combined in a 1:1 ratio (1 µM), followed by a 5 min incubation at room temperature for dRNPC assembly at a final concentration of 500 nM. The dRNPC was diluted to the indicated concentrations when needed.

**TABLE 1:** Alt-R CRISPR-Cas9 crRNA.

gRNA name	Alt-R CRISPR-Cas9 crRNA
gRNA-BbsI	/AlTR1/rGrUrArGrArArGrArCrArArArUrCrCrArUrGrUrArGrUrUrUrUrArGrArGrCrUrArUrGrCrU/AlTR2/
gRNA-XbaI	/AlTR1/rCrUrArGrArArArGrUrGrArArCrUrCrGrUrArArUrGrUrUrUrUrArGrArGrCrUrArUrGrCrU/AlTR2/

### DNA binding by *in vitro* assembled dCas9-sgRNA RNP complex

Reactions were performed in 20 µL volumes using various amounts of target DNA ranging from 50 to 200 ng, 2 µL of the specific buffer for either single or dual restriction enzymes, nuclease-free water, and without or with different concentrations of dRNPCs ranging from 50 nM to 400 nM. The total reaction was incubated at 37°C for various incubation periods ranging from 5 to 60 min to allow the binding of the dRNPC onto the DNA.

### Cleavage assays

Restriction enzymes were added to the total reaction at a final concentration of 1 U/µL, and then, the mixtures were incubated at 37°C for various times. After incubation, 4 μL of 6X loading dye was added to the reaction. The products of each reaction were assessed by electrophoresis on 1.5% agarose gel pre-stained with SYBR-safe dye (Thermo Fisher Scientific, United States) and using 1 Kb plus GeneRuler DNA ladder at a ratio of 1:50,000 dilution (Thermo Fisher Scientific, United States).

### PCR cloning of the M gene in pcDNA3.1

The M gene was amplified from the pIDTSMARTAmp-M synthetic plasmid DNA by PCR using the following pair of primers, M-*BamHI*-F and M-*EcoRI*-R (Table 2), using a BioRad C1000 Touch thermal cycler (40 cycles) and the AccuPrimeTM *Taq* DNA Polymerase, High Fidelity (Thermo Fisher Scientific, Cat. No. 12346-094). The QIAquick PCR purification kit was used to purify the amplified DNA fragment and was quantified by NanoDrop. The restriction endonucleases (*BamHI*-HF and *EcoRI*-HF) were used for the restriction digestion of the purified M gene and pcDNA3.1. The purified digested products of the insert (M gene) and the vector (pcDNA3.1) were then mixed together with T4 DNA ligase (1U/μL) using various molar ratios of the insert genes to the vectors (1:1, 1:3, 1:6, and 3:1) and incubated 18 h at 16°C for the ligation reaction. One Shot TOP10 competent *E. coli* cells were transformed with 2 μL of the ligation reaction and plated onto LB agar plates with 100 μg/mL of ampicillin. Positive clones were isolated, amplified, and confirmed for the presence of inserts by Sanger sequencing using a pair of primers, pcDNA3.1-F and pcDNA3.1-R (Table 2), as described previously (Saifaldeen et al., 2021).

**TABLE 2:** Primers used in this study.

Primer name	Primer sequence (5′–3′)	Used to
M1-*BbsI*-F	atg​gca​gat​tcc​aac​ggt​act​att​acc​gtt	Amplify the M1 fragment
M1-*BbsI*-R	cct​aca​aga​caa​gcc​att​gcg​ata​gca​att	Amplify the M1 fragment
M2-*XbaI*-F	ttg​tag​gct​tga​tgt​ggc​tca​gct​act​tca	Amplify the M2 fragment
M2-*XbaI*-R	ttt​ctt​tag​gca​ggt​cct​tga​tgt​cac​agc	Amplify the M2 fragment
M-*BbsI*-*XbaI*-F	atg​gca​gat​tcc​aac​ggt​act​att​acc​gtt	Amplify the M gene
M-*BbsI*-*XbaI*-R	tta​ctg​tac​aag​caa​agc​aat​att​gtc​act​gct​a	Amplify the M gene
M-BamHI-pcDNA3.1-F	gga​tcc​atg​gca​gat​tcc​aac​ggt​act​att​acc	Amplify the M gene and clone it in pcDNA.3.1
M-EcoRI-pcDNA3.1-R	gaa​ttc​tta​ctg​tac​aag​caa​agc​aat​att​gtc​act​gct	Amplify the M gene and clone it in pcDNA.3.1
pcDNA3.1-F	att​tcc​aag​tct​cca​ccc​cat​tg	Sequence the M gene in pcDNA3.1
pcDNA3.1-R	agg​aca​gtg​gga​gtg​gca​cc	Sequence the M gene in pcDNA3.1

### Cell culture media, conditions, and DNA transfections

HEK293T cells were cultured in Dulbecco’s Modified Eagle Medium (DMEM) supplemented with 10% fetal bovine serum in a 37°C humidified incubator with 5% CO2. Transient transfection of cells was performed with FuGene HD DNA Transfection Reagent following the manufacturer’s instructions (Bio-Rad, Hercules, CA, United States). In brief, 320,000 HEK293T cells per well were seeded in a six-well culture plate and cultured in DMEM supplemented with 2 mM fresh l-glutamine 24 h prior to transfection. pcDNA3.1-CMV-M (2 μg) were transfected in HEK293T cells using 13.6 μL of FuGene HD, and cells were incubated for 48 h at 37°C in a humidified incubator with 5% CO_2_.

### Reverse transcriptase-recombinase polymerase amplification (RT-RPA)

Transfected HEK293T cells expressing the M gene were harvested for total RNA extraction using the RNeasy Mini Kit (QIAGEN Cat. No. 74104) following the manufacturer’s instructions. Total RNA was eluted in DNase- and RNase-free water and quantified by NanoDrop. RT-RPA was performed using a set of primers (Table 2) to amplify the SARS-CoV-2 M gene as separate fragments (M1 and M2 for targeting *BbsI* and *XbaI*, respectively) or the full length M gene containing both *BbsI* and *XbaI* sites. RT-RPA reaction was set up as recommended in the TwistAmp^®^ Basic kit (TwistDx, UK). A total of 10 ng of RNA was derived from the transfected HEK293T cells and the individual patient samples, and the reaction was performed at 39°C for 10 min using the primers indicated in Table 2 to amplify M1, M2, and M-DNA fragments. Amplicons were then purified with a QIAGEN PCR clean up kit (QIAGEN Cat. No. 28106) following the manufacturer’s instructions. DNA was eluted in 40 μL of DNase- and RNase-free water quantified by NanoDrop and visualized on agarose gel. The desired amount of the DNA target was used for the *in vitro* dCPRES assay.

## Results and discussion

### Increasing concentrations of dRNPCs block RE cleavage of the M gene

**FIGURE 1:**
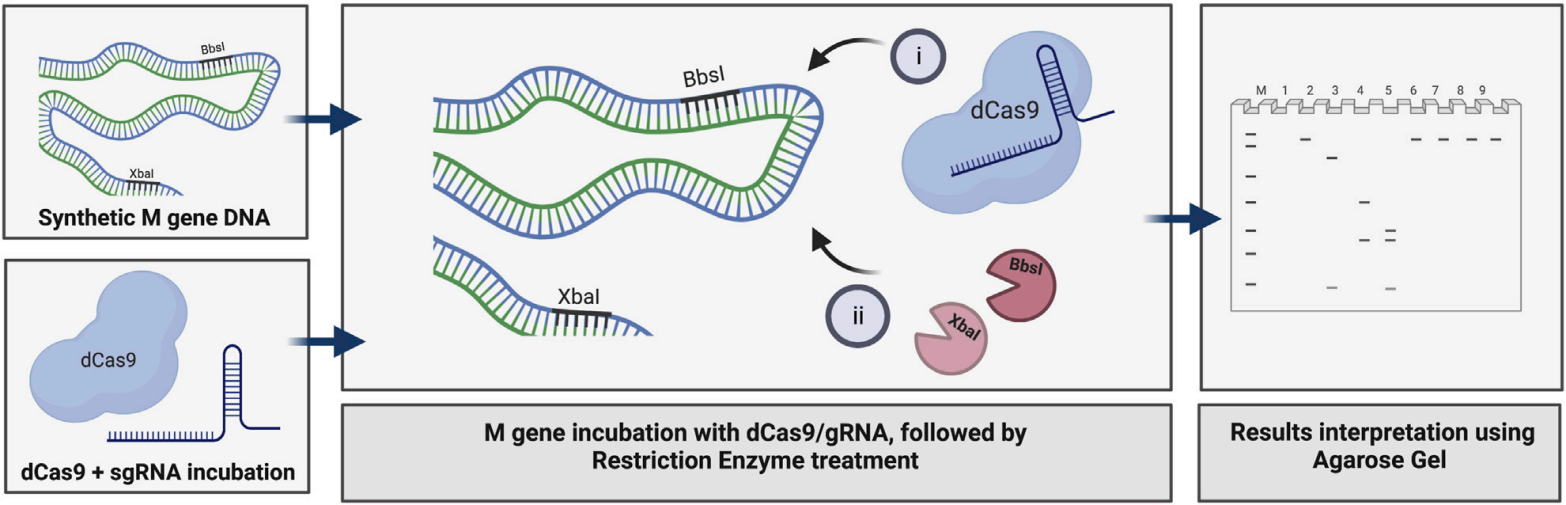
Principle of dCas9-sgRNAs complexes in binding specifically to the SARS-CoV-2 M gene and inhibiting the restriction enzyme activities of *BbsI* and *XbaI*. The amplified synthetic M gene is incubated with complexes of dCas9-sgRNA targeting *BbsI* and *XbaI* restriction sites to block RE cleavage. The inhibition of both restriction enzymes activity is assessed using agarose gel electrophoresis, created with BioRender.com.

**FIGURE 2:**
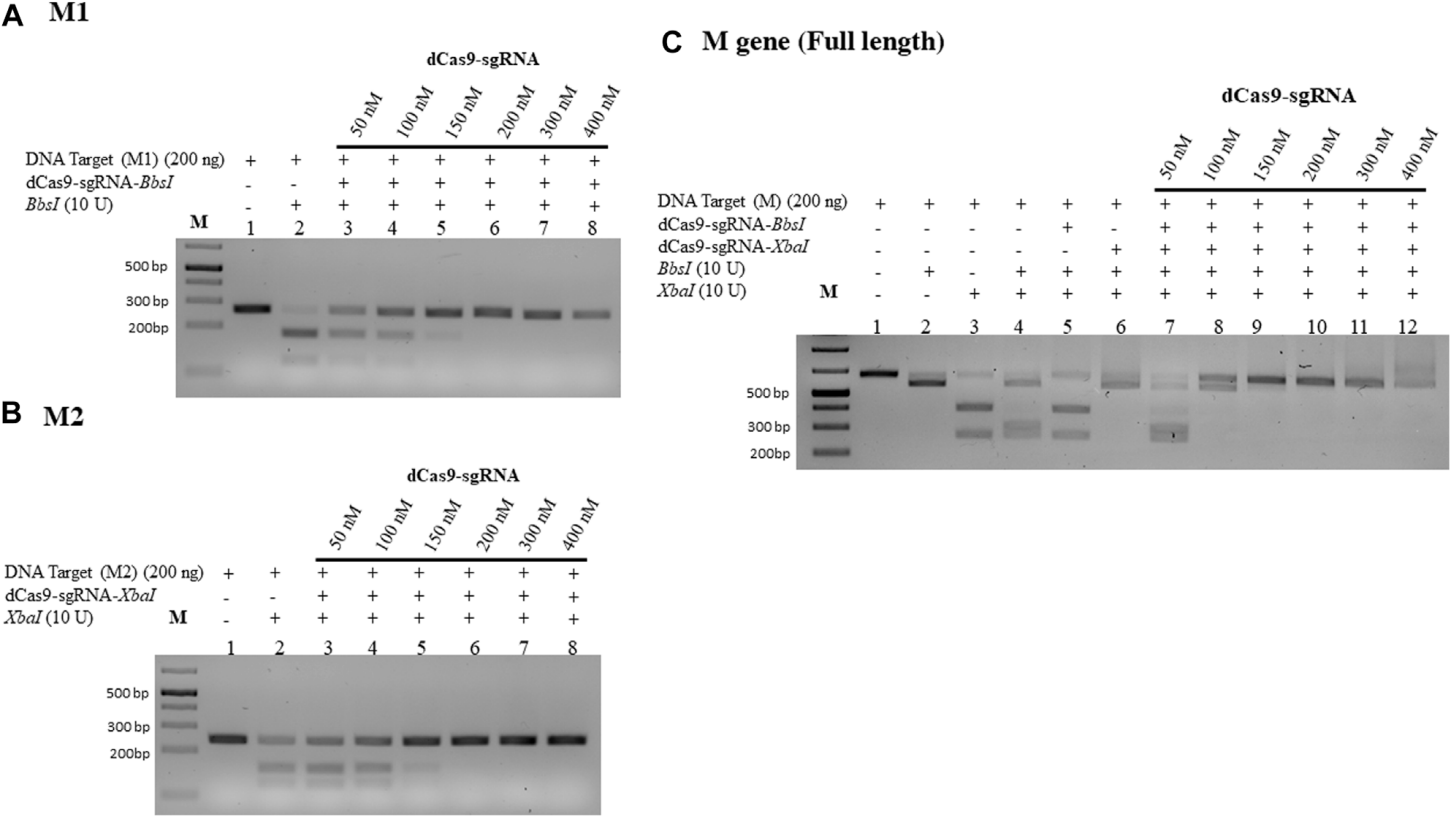
Increasing concentrations of dCas9-sgRNAs complexes block the activities of *BbsI* and *XbaI* restriction enzymes. **(A)** 200 ng of PCR fragment 1 of the M gene (M1) subjected to *BbsI* restriction enzyme digestion without or with pre-incubation with increasing concentrations of dCas9-sgRNA targeting the *Bbs*I restriction site. **(B)** 200 ng of PCR fragment 2 of the M gene (M2) subjected to *XbaI* restriction enzyme digestion without or with pre-incubation with increasing concentrations of dCas9-sgRNA targeting the *Xba*I restriction site. **(C)** 200 ng of PCR fragment of the full-length M gene subjected to single or dual restriction enzyme digestion (*BbsI or/and XbaI)* without or with pre-incubation with increasing concentrations of dCas9-sgRNA-*BbsI*, dCas9-sgRNA-*XbaI*, or multiplexing of both. Restriction enzyme site blocking efficiencies by the dCas9-sgRNAs are interpreted from the diminishing release of fragment(s) following digestion. M: DNA marker 1 Kb plus GeneRuler. Samples were run on 1.5% agarose gel stained with SYBR-safe dye (2 µL of 10,000 X concentrate (Invitrogen).

We used the M gene of the virus as the target to develop a diagnostic protocol using the dRNPC approach (Figure 1). To carry out the analysis, we designed two sgRNAs to specifically block the RE sites, *BbsI* and *XbaI*, which are uniquely present in the M gene (Figure 1; Supplementary Figure S1). In this experiment, we amplified either the M gene or two separate fragments, M1 or M2, of the M gene (Supplementary Figure S1). The *in vitro* assay used a fixed amount of 200 ng of the DNA target fragment (either the M1 fragment bearing the *BbsI* site, the M2 fragment bearing the *XbaI* site, or the synthetic M gene bearing both RE sites) in the presence of increasing concentrations of dRNPCs to monitor the DNA cleavage. The dRNPCs carried sgRNAs that can bind to either *BbsI* or *XbaI* sites of the M1 and M2 fragments, respectively (Supplementary Figure S1). In the absence of the dRNPC, *BbsI* cleaved the M1 fragment to release the specific digested fragments (175 and 91 bp) (Figure 2A). Likewise, in the absence of dRNPC, *XbaI* cleaved the M2 fragment to release the specific digested fragments (140 and 103 bp) (Figure 2B). In addition, in the absence of dRNPCs, both *BbsI* and *XbaI* cleaved the M gene to release three specific fragments (308, 103, and 91 bp) (Figure 2C). However, in the presence of increasing amounts of the dRNPCs (ranging from 50 to 400 nM), it progressively blocked the RE activities of either *BbsI* (Figure 2A, lane 3–8), *XbaI* (Figure 2B, lane 3–8) or both *BbsI* and *XbaI* (Figure 2C, lanes 7–12) from cleaving the M1, M2, or the M gene, respectively. Complete inhibition of the RE was observed starting from 200 nM of the dRNPCs (Figure 2A lane 6, 2B lane 6, and 2C lane 10, respectively). These data strongly suggest that this approach can be used to specifically block the RE sites of the viral M gene using specific sgRNAs targeting the restriction enzyme sites.

### dRNPCs effectively protect lower amounts of M-DNA from RE cleavage

**FIGURE 3:**
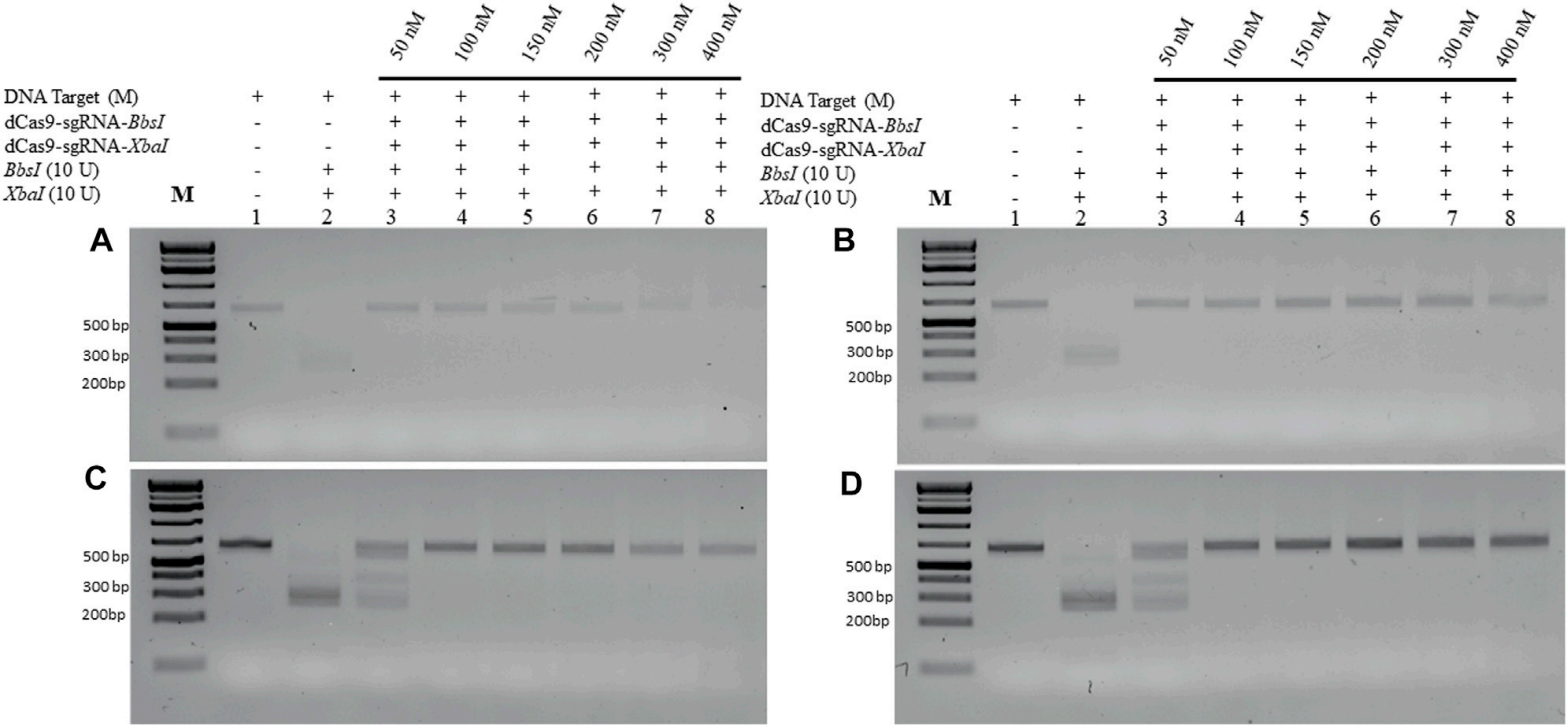
Increasing amount of M-DNA target is protected from RE cleavage by dCas9-sgRNAs complexes. Increasing amounts of the M-DNA target **(A)** 25 ng, **(B)** 50 ng, **(C)** 75 ng, and **(D)** 100 ng were subjected to dual restriction enzyme digestion, *BbsI* and *XbaI*, without or with pre-incubation with increasing concentrations of multiplexing dCas9-sgRNA-Xba and dCas9-sgRNA-*XbaI,* followed by restriction enzyme cleavage for 1 h at 37°C. Restriction enzyme site blocking efficiencies by the dCas9-sgRNAs are interpreted from the diminishing release of fragment(s) following digestion. M: DNA marker 1 Kb plus GeneRuler.

We next examined the lowest detectable amount of the M-target DNA that can be protected by the dRNPCs. In this analysis, we used a range of the M-target DNA from 25 to 100 ng with increasing amounts of the dRNPCs from 50 to 400 nM (Figure 3). Although the bands are very faint (because SYBR-safe can barely detect low amounts of DNA), we observed that 50 nM of the dRNPCs were sufficient to protect 25–50 ng of the M-target DNA from cleavage by both REs, *BbsI,* and *XbaI* (Figures 3A, B, lane 3 vs. 2). We chose to use 75 ng of the M-target DNA and 100 nM of the dRNPCs for the remaining experiments for better visualization on the SYBR-safe agarose gel (see Figure 3C, lane 4). From our experience, we believe that this assay can be adapted to use a much lower amount of DNA depending on the detection system available for DNA, such as using fluorescently labeled DNA.

### dRNPCs rapidly protect the M-DNA from cleavage by the restriction enzymes

**FIGURE 4:**
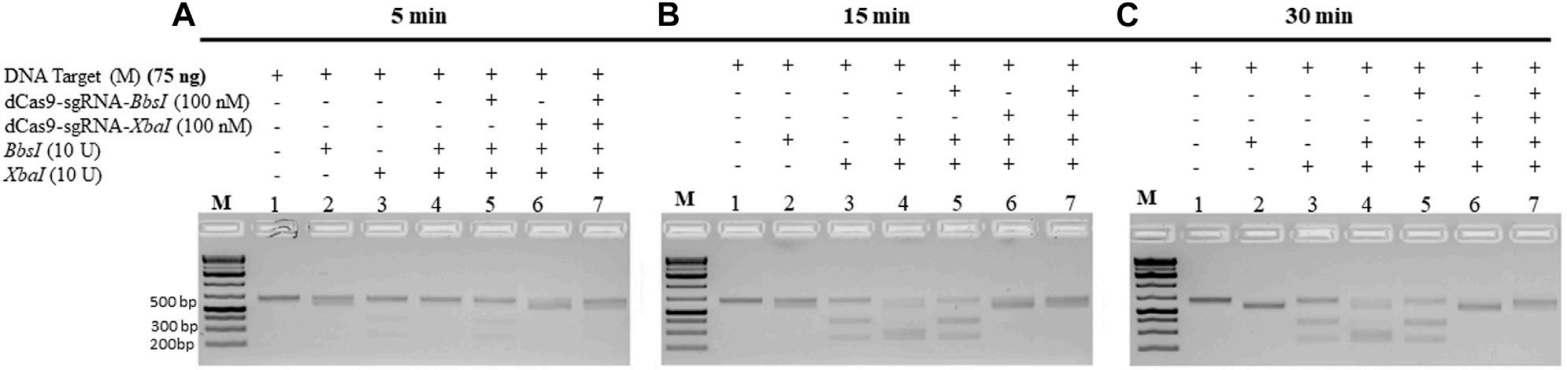
dCas9-sgRNAs complexes rapidly protect the M gene from cleavage by the restriction enzymes. The entire M gene (75 ng) was pre-incubated for **(A)** 5 min, **(B)** 15 min, and **(C)** 30 min, with increasing concentrations of multiplexing dCas9-sgRNA-*BbsI* and dCas9-sgRNA-*XbaI*, followed by restriction enzyme cleavage for 1 h at 37°C. Restriction enzyme site blocking efficiencies by the dCas9-sgRNAs are interpreted from the diminishing release of fragment(s) following digestion. M: DNA marker 1 Kb plus GeneRuler.

The abovementioned experiments were conducted under conditions when the dRNPCs were incubated with the M-target DNA for 1 h. We next checked the minimum time required by the dRNPCs to bind to the M-target DNA and block the activities of the REs. The data revealed that the dRNPCs were able to bind to the DNA within 15 min and protected the M-target from the RE cleavage (Figure 4B, lane 7 vs. lane 4), indicating that binding of the dRNPCs to the DNA is rapid. We expect that this rapid effect would be independent of the protected DNA, ensuring that this tool can be applied to other targets.

### Increasing concentrations of the REs did not interfere with the protection of the M-DNA by the dRNPCs

**FIGURE 5:**
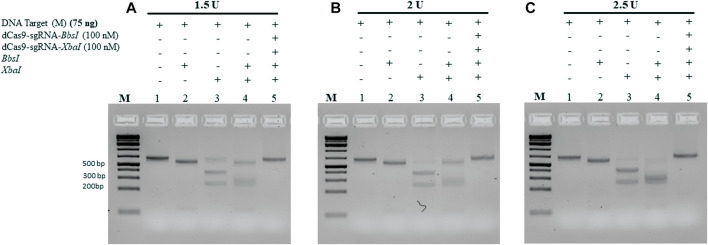
Increasing the concentration of the restriction enzymes *BbsI* and *XbaI* did not interfere with the protection of the M-DNA by the dCas9-gRNA complexes. A total of 75 ng of the M gene was pre-incubated for 5 min with the dCas9-sgRNAs complexes, followed by 15 min incubation with the restriction enzymes *BbsI* and *XbaI* reaction at **(A)** 1.5 U, **(B)** 2 U, and **(C)** 2.5 U. Restriction enzyme site blocking efficiencies by the dCas9-sgRNAs are interpreted from the diminishing release of fragment(s) following digestion. M: DNA marker 1 Kb plus GeneRuler.

All the aforementioned experiments were conducted using 10 units of each RE and incubated for 1 h. To determine the minimum amount of RE required to cleave 75 ng of the M-target DNA, we pre-incubated the M-target DNA in the absence and presence of 100 nM of the dRNPCs for 15 min followed by incubation with varying amounts of the REs. As shown in Figure 5A, 1.5 units of each enzyme were sufficient to cleave the M-target DNA but were completely blocked by the dRNPCs (lane 4 vs 5). Excess of the RE, that is, 2 and 2.5 units, did not interfere with the function of the dRNPCs (Figures 5B, C, respectively). Based on the agarose gel analysis, the data strongly suggest that the experiments can be conducted with i) 75 ng of target DNA, ii) 100 nM of dRNPCs pre-incubated for 15 min, and iii) digestion with 1.5 units of REs for 15 min (Figure 5). As such, we referred to this entire protocol as dead Cas9 Protects Restriction Enzyme Sites (dCPRES).

### dCPRES specifically detects the M gene expressed in HEK293 cells and from individuals tested positive by RT-qPCR

**FIGURE 6:**
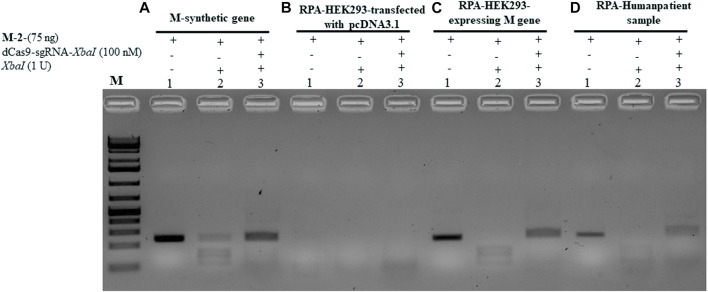
dCPRES specifically detects the M gene expressed in HEK293 cells and from individuals tested positive by RT-qPCR. A total of 75 ng of M-2 fragment derived from **(A)** the synthetic M gene or **(B)** RT-RPA of RNA samples extracted from HEK293 cells transfected with pcDNA3.1 vector or **(C)** RT-RPA of RNA samples extracted from HEK293 cells expressing the M gene or **(D)** an individual tested positive by RT-PCR. All fragments were subjected to single *XbaI* restriction enzyme digestion without or with pre-incubation, with increasing concentrations of dCas9-sgRNA targeting *XbaI* restriction site. Restriction enzyme site blocking efficiencies by the dCas9-sgRNAs are interpreted from the diminishing release of fragment(s) following digestion. M: DNA marker 1 Kb plus GeneRuler.

To validate the aforementioned dCPRES approach, we expressed the M gene in human HEK293 cells using the pcDNA3.1 expression system that drives expression from the CMV promoter. Total RNA was isolated from these cells and reverse transcribed using reverse polymerase amplification (RPA), followed by the detection of the M-2 fragment of the M gene using the dCPRES protocol. The data revealed that the M-2 fragment was amplified from the HEK293 cells to the same extent as the synthetic M-2 fragment (Figure 6C, lane 1 vs. Figure 6A, lane 1), suggesting that the M gene can be detected *in vivo*. The amplified M-2 fragment was efficiently cleaved by the *XbaI* restriction enzyme (Figure 6C, lane 2). Importantly, binding of the dRNPC (dCas9-sgRNA-*XbaI*) to the M-2 fragment completely blocked the *XbaI* cleavage site (Figure 6C, lane 3). This analysis indicates that the amplification of the endogenous M gene, followed by its protection from cleavage by the dRNPC, provides a highly specific way to detect the viral gene *in vivo*.

We next took total RNA isolated from the nasal sample from SARS-CoV-2-positive individuals determined by RT-qPCR and examined for the expression and protection of the M-2 fragment (see Materials and Methods). As shown in Figure 6D, lane 1, the M-2 fragment was amplified from the positive individual and completely protected from *XbaI* cleavage when bound by dRNP (dCas9-sgRNA-*XbaI*) (Pane D, lane 3). To further validate this approach, we obtained another nine clinical samples with different CT values ranging from 15 to 28. In all the cases, the M-2 gene fragment was amplified using RPA (see Supplementary Figure S2, lane 1 for samples 1–1 to 1–9) and protected by the RNP complex from *XbaI* digestion (lane 3 for samples 1–1 to 1–9). We believe that the conditions for detecting the M-2 fragment would be the same as for detecting the M-1 or the M gene, although we did not follow the amplification of the M-1 or the M gene.

## Conclusion

**FIGURE 7:**
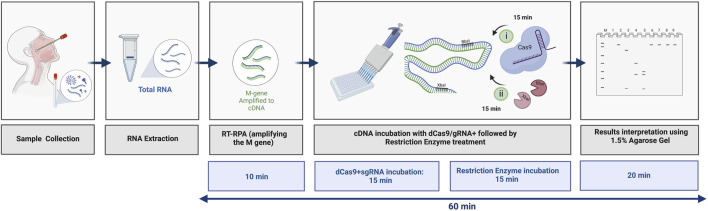
Illustrative diagram of the different steps in using the dCRPES kit to detect the SARS-Cov-2. The same illustration used in Figure 1 but with the timing required for each step to detect the SARS-Cov-2 upon receiving the RNA samples, created with BioRender.com.

In summary, we have developed a rapid and precise protocol that is based on the dead Cas9-sgRNA complex in protecting REs, specifically from cleaving the M gene of SARS-CoV-2. As shown in Figure 7, the total time required to detect the M gene using the dCPRES protocol from extracted RNA is 60 min. The dCPRES method consisted of four steps: i) the RT-RPA step required 10 min, ii) the incubation of the cDNA with dRNPCs needed 15 min, iii) the restriction enzyme digestion for 15 min, and iii) the result interpretation by agarose gel required 20 min (Figure 7). However, the protocol does not take into consideration the time needed to extract the total RNA.

Herein, our novel dCPRES approach is a proof of concept that can be used to detect viral infectious diseases and pathogens at the nucleic acid level. In the case of SARS-CoV-2, our approach is based on three layers of specificity that will lead to the positive identification of the virus and using primarily the M gene, as we have not selected to test another unique region of the SARS-CoV-2 genome. Compared to RT-qPCR, our dCPRES approach has the advantage that it is rapid (that is, 60 min vs. the average time of 120 min for RT-qPCR) and specific in that it depends on the M gene, which is essential.

**FIGURE 8:**
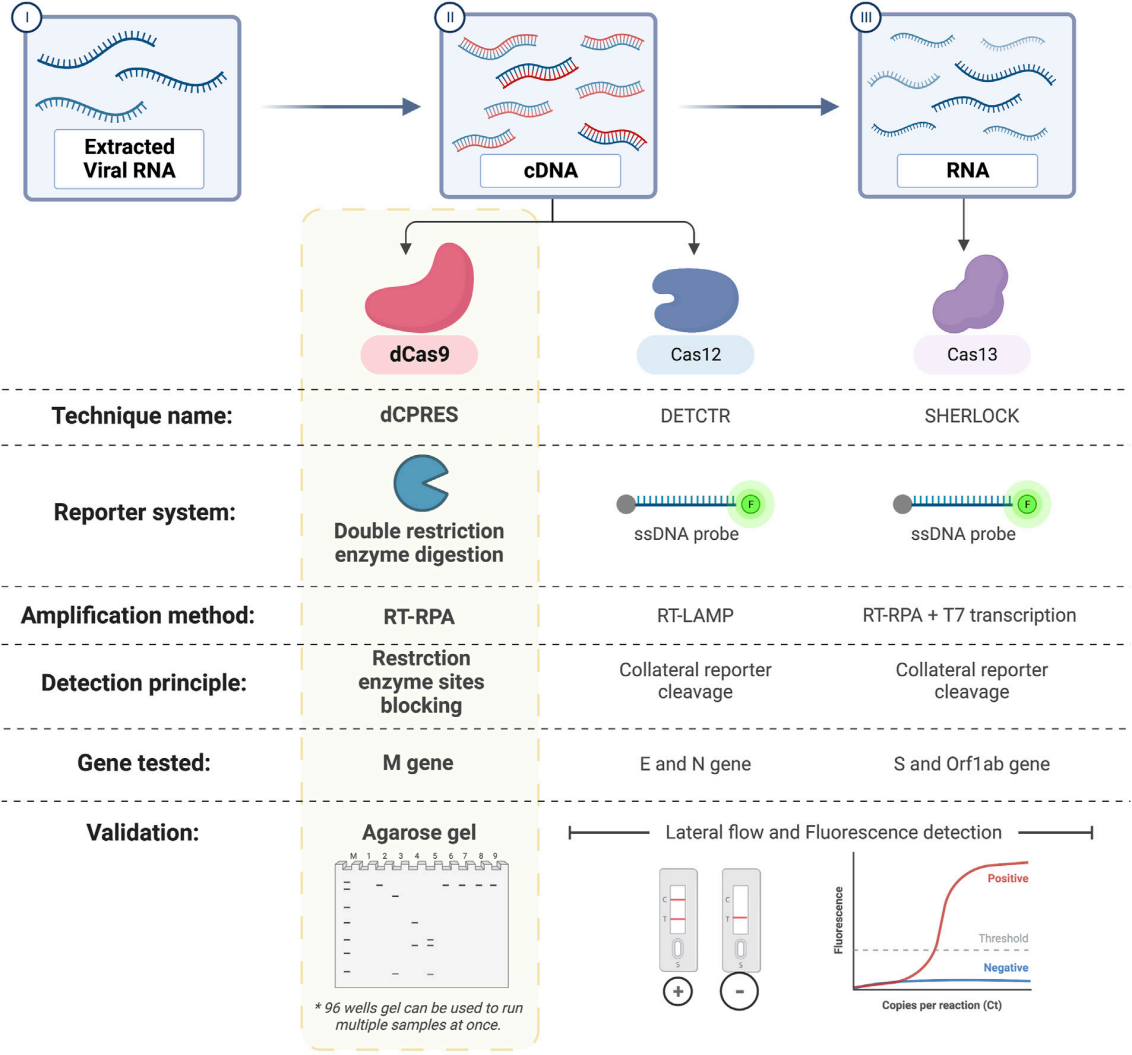
Comparison between dCPRES and other CRISPR-based techniques used to detect SARS-Cov-2. dCRPES has the advantage that it includes an additional specific step, that is, the RE cleavage sites are blocked, as compared to other CRISPR-based diagnostic platforms such as Cas12 and Cas13 designed to the detect the E and N gene, and the S gene of SARS-CoV-2, created with BioRender.com.

Compared to other CRISPR-based techniques, our dCPRES approach includes an additional specific step, as shown in Figure 8; that is, the RE cleavage sites are blocked, whereas other CRISPR-based diagnostic platforms such as Cas12 and Cas13 are designed to detect the E, N, and S genes of SARS-CoV-2 in a single-step approach. The limitations of using the dCPRES approach for molecular diagnostics to detect viruses and pathogens such as fungi are i) finding a suitable NGG PAM sequence that is required for the binding of the RNP to the target DNA sequence and block the REs and ii) the absence of a unique restriction enzyme site in the target DNA.

## Data Availability

The raw data supporting the conclusions of this article will be made available by the authors, without undue reservation.

## Supplementary material

SUPPLEMENTARY FIGURE S1 Illustration of the entire M-gene, the position of the targeted gRNA, the position of the targeted restriction sites by BbsI (in RED) and XbaI (in GREEN) in the M1 and M2 regions, respectively, and in the full length M gene.

SUPPLEMENTARY FIGURE S2 dCPRES detects specifically the M gene expressed from 9 individuals tested positive by RT-qPCR. 75 ng of M-2 fragment derived from nine tested positive individuals (I-1 to I-9).
