# Supplementary material for: A CRISPR-based approach using dead Cas9-sgRNA to detect SARS-CoV-2
Source: Front Mol Biosci. 2023 Jun 14;10:1201347. doi: 10.3389/fmolb.2023.1201347 (PMC10300348; doi:10.3389/fmolb.2023.1201347)
Supplement: Supplementary file 1 [file Table1.DOCX]

**Supplementary Figures**

**
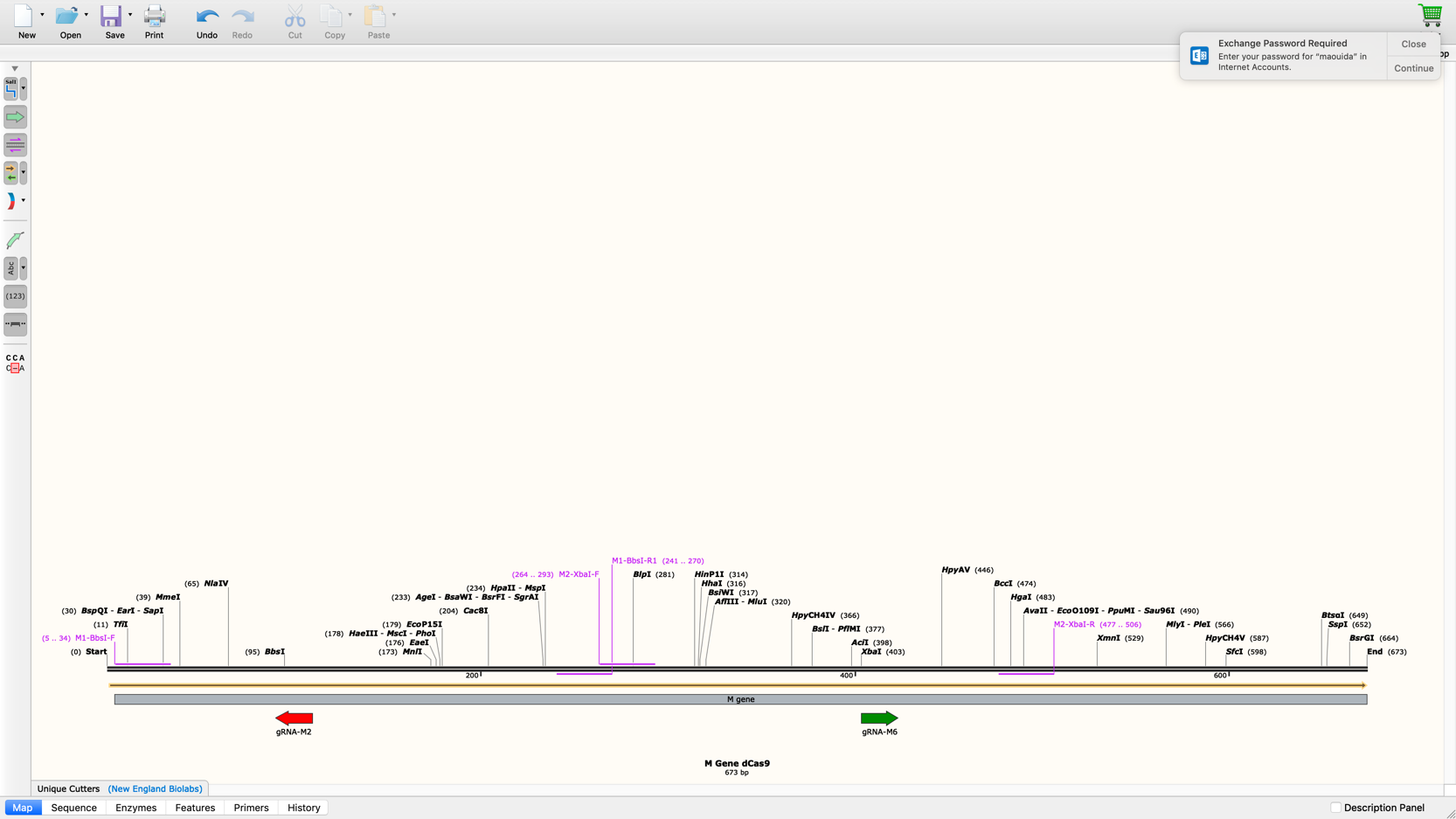
**

**
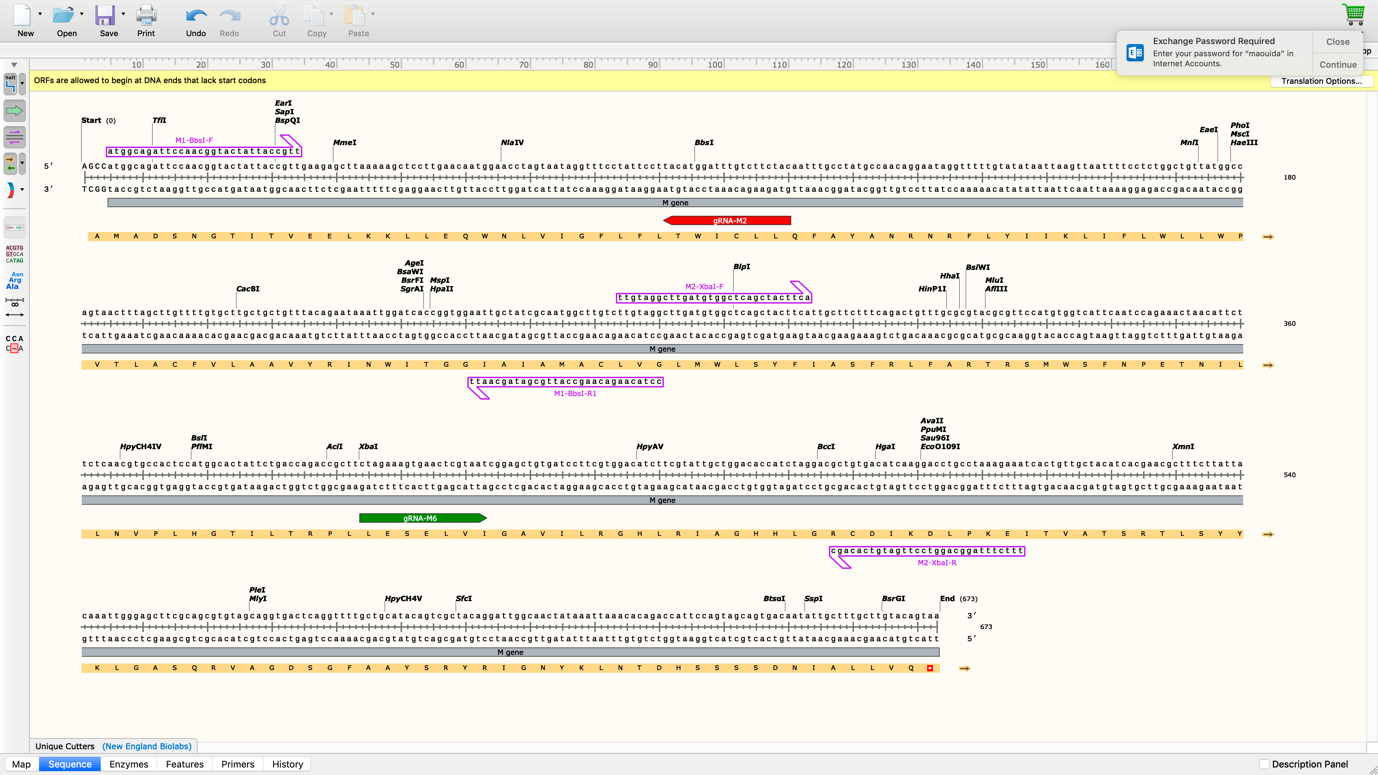
**

**Supplementary Fig. S1 :** Illustration of the entire M-gene, the position of the targeted gRNA, the position of the targeted restriction sites by BbsI (in RED) and XbaI (in GREEN) in the M1 and M2 regions, respectively, and in the full length M gene.

**
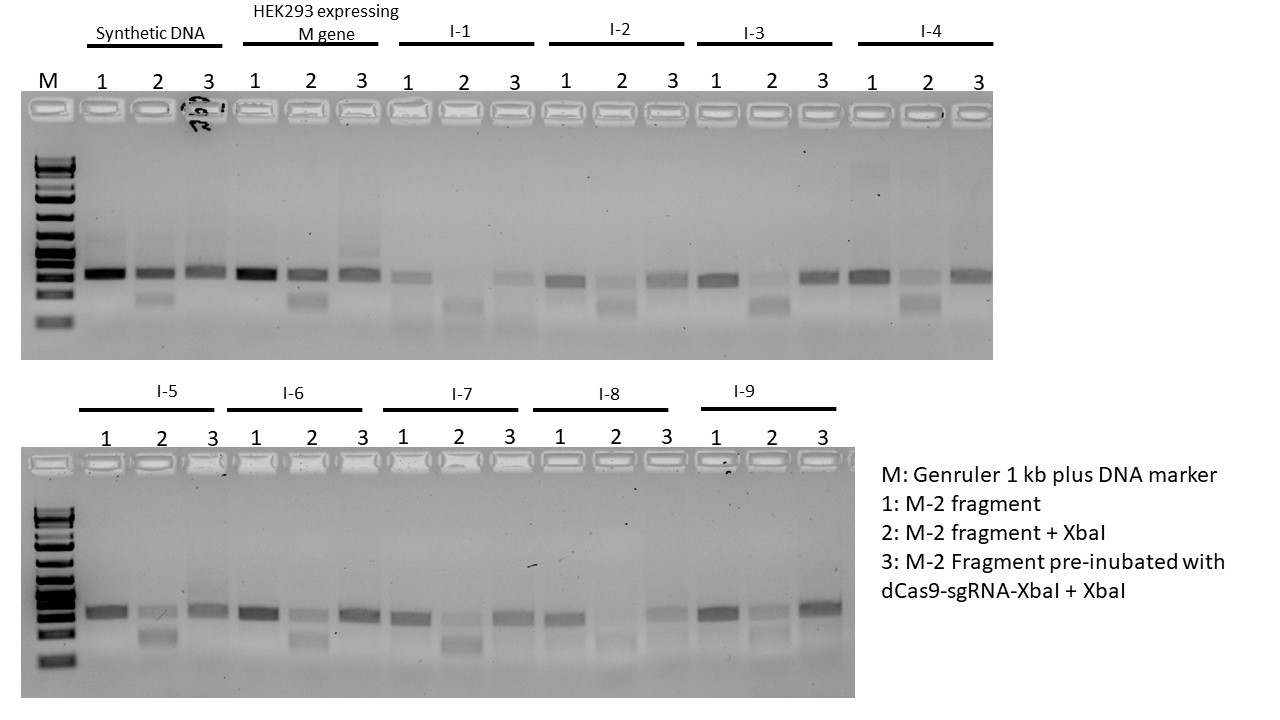
**

**Supplementary Fig. S2: dCPRES detects specifically the M gene expressed from 9 individuals tested positive by RT-qPCR.** 75 ng of M-2 fragment derived from nine tested positive individuals (I-1 to I-9).
