# Supplementary figures and images for: A CRISPR-based approach using dead Cas9-sgRNA to detect SARS-CoV-2
Source: Front Mol Biosci. 2023 Jun 14;10:1201347. doi: 10.3389/fmolb.2023.1201347 (PMC10300348; doi:10.3389/fmolb.2023.1201347)

## Slide 1
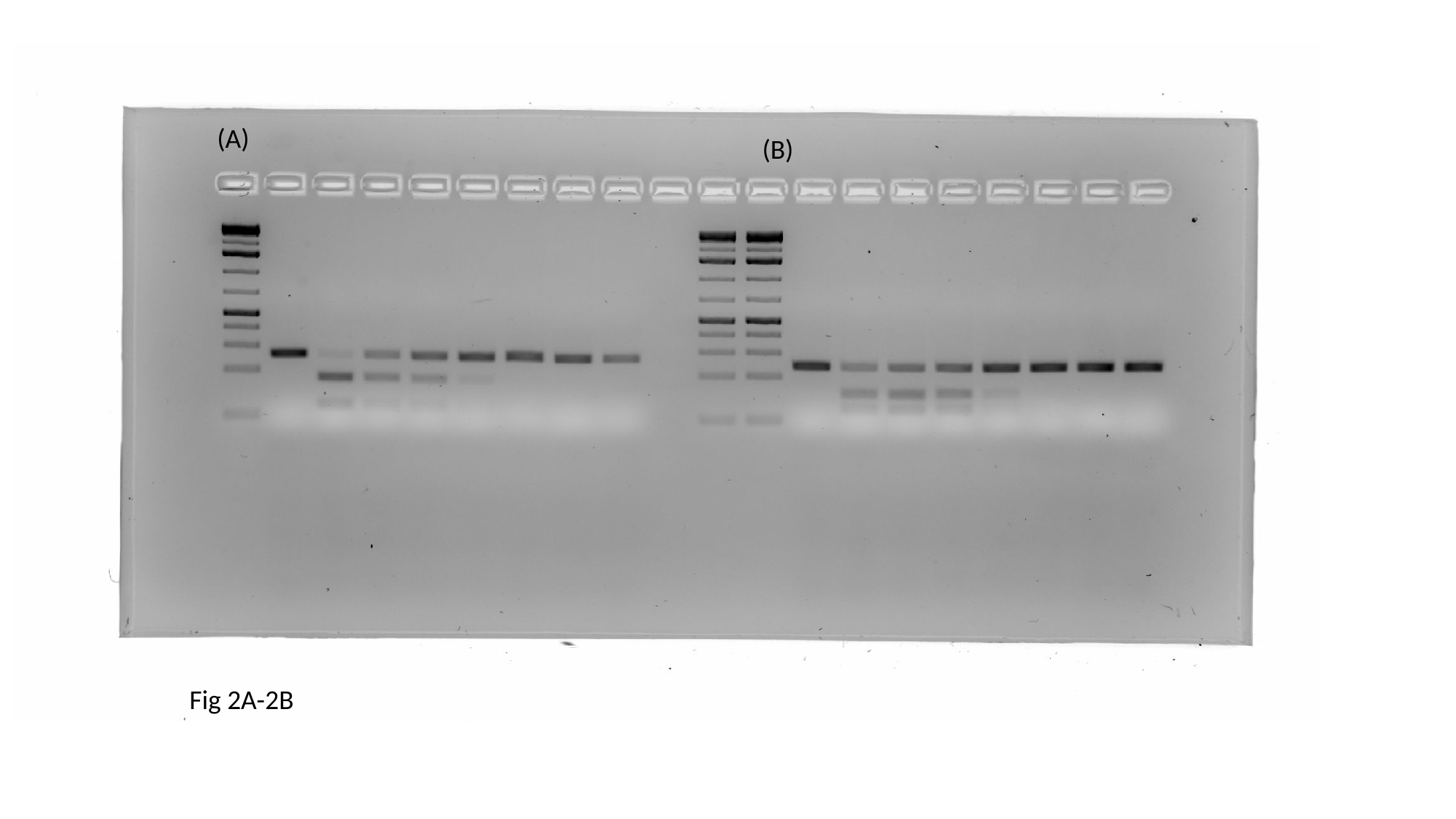

(A)
(B)
Fig 2A-2B

## Slide 2
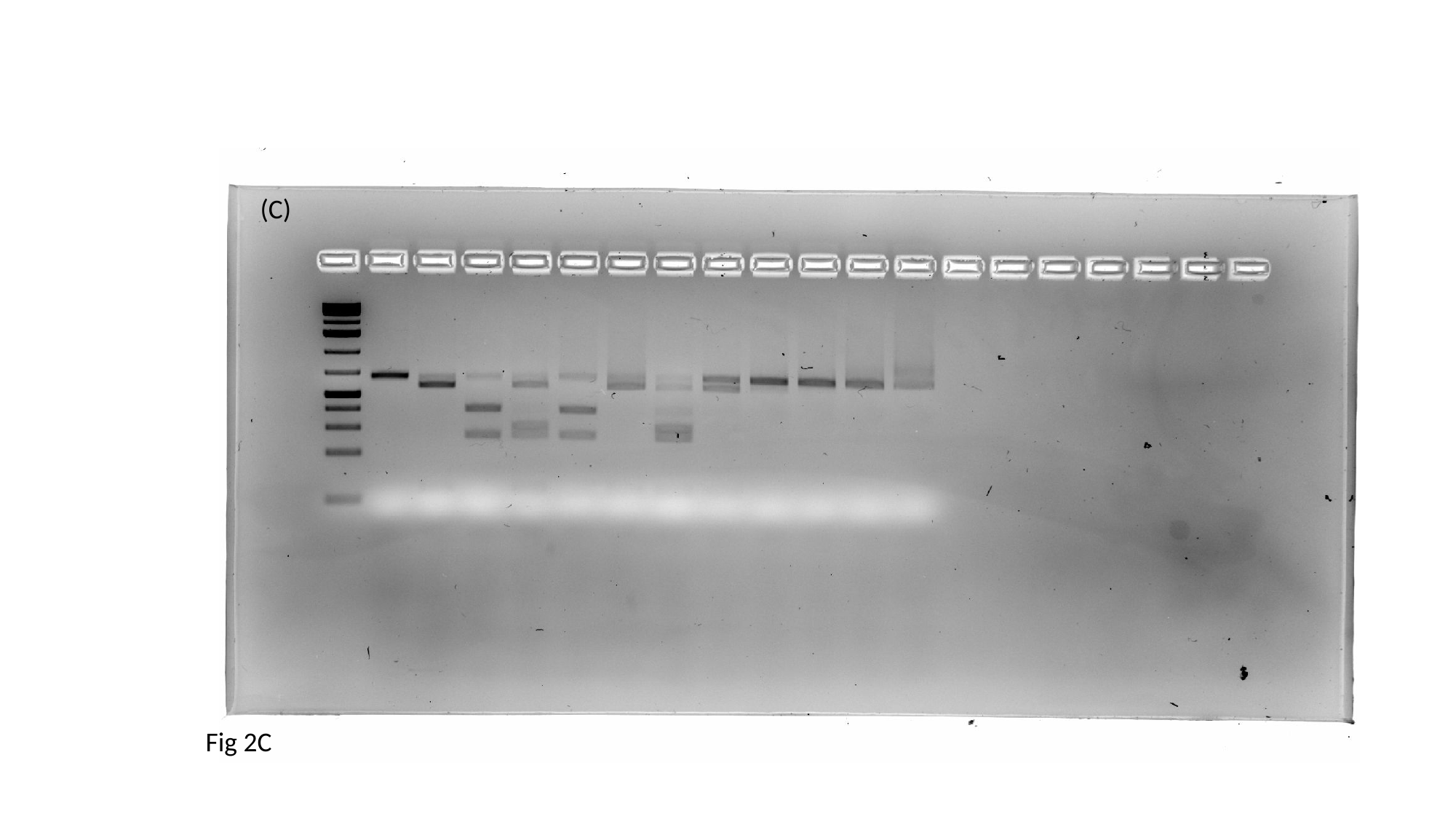

(C)
Fig 2C

## Slide 3
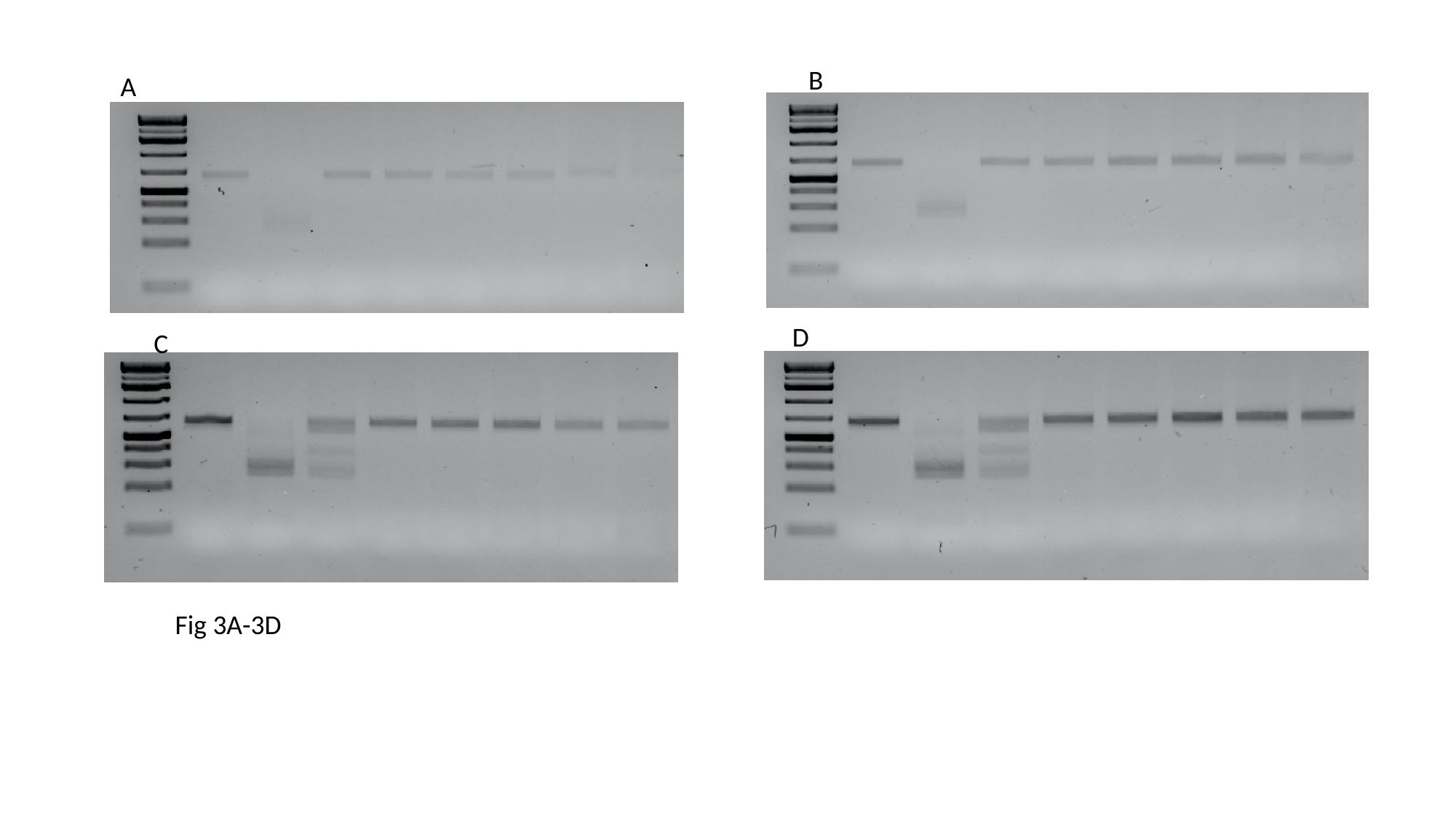

B
A
D
C
Fig 3A-3D

## Slide 4
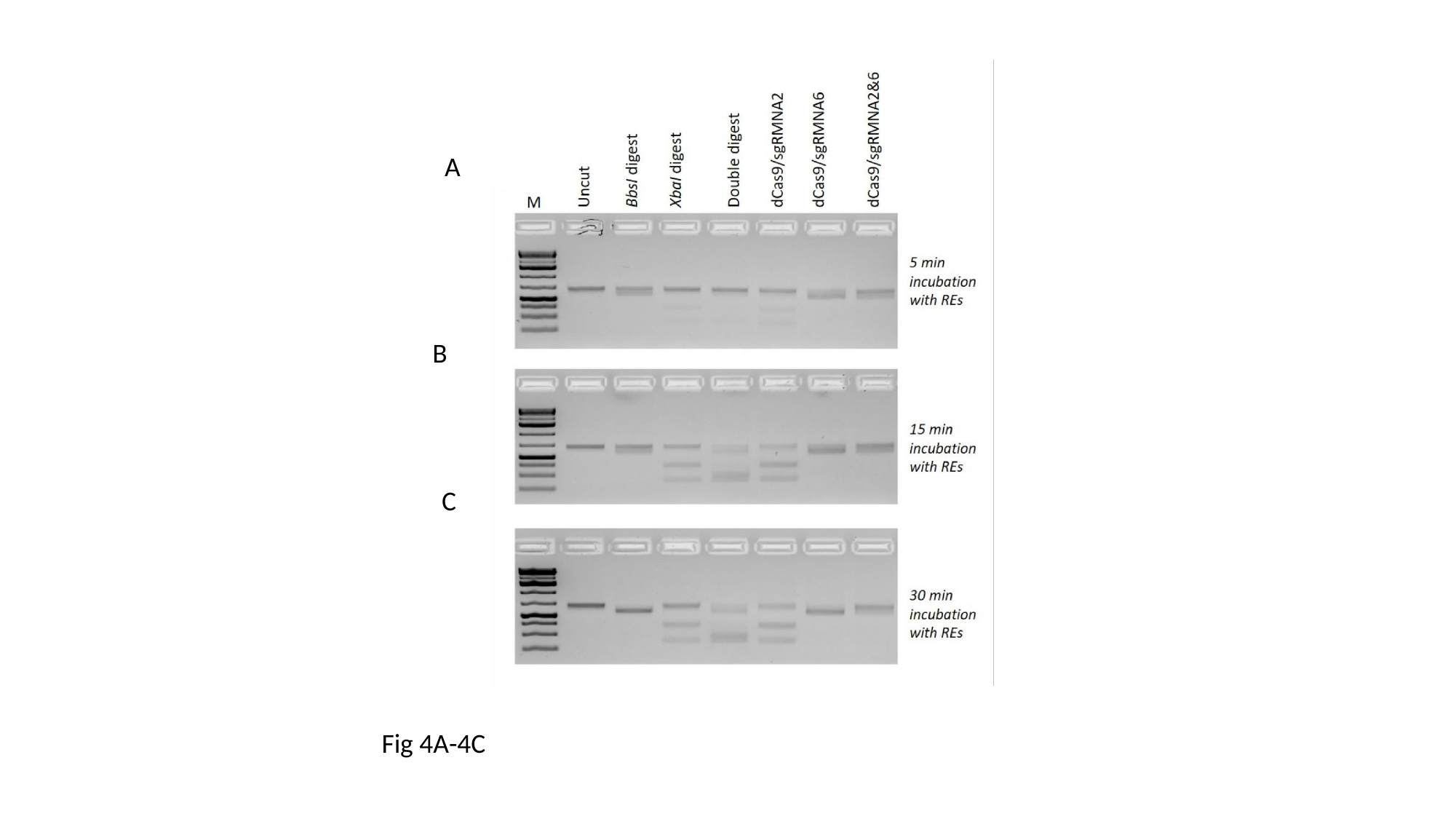

A
B
C
Fig 4A-4C

## Slide 5
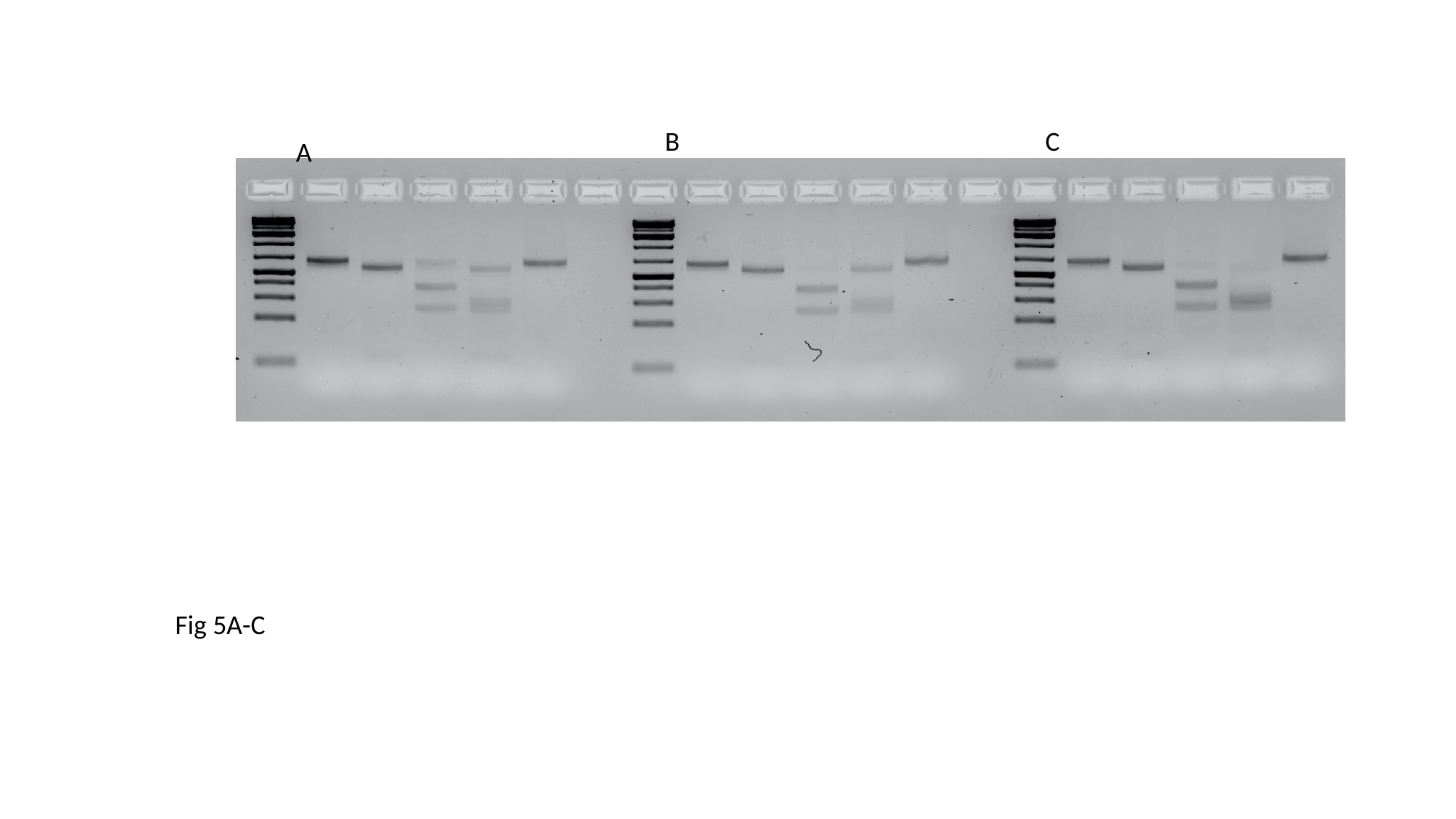

B
C
A
Fig 5A-C

## Slide 6
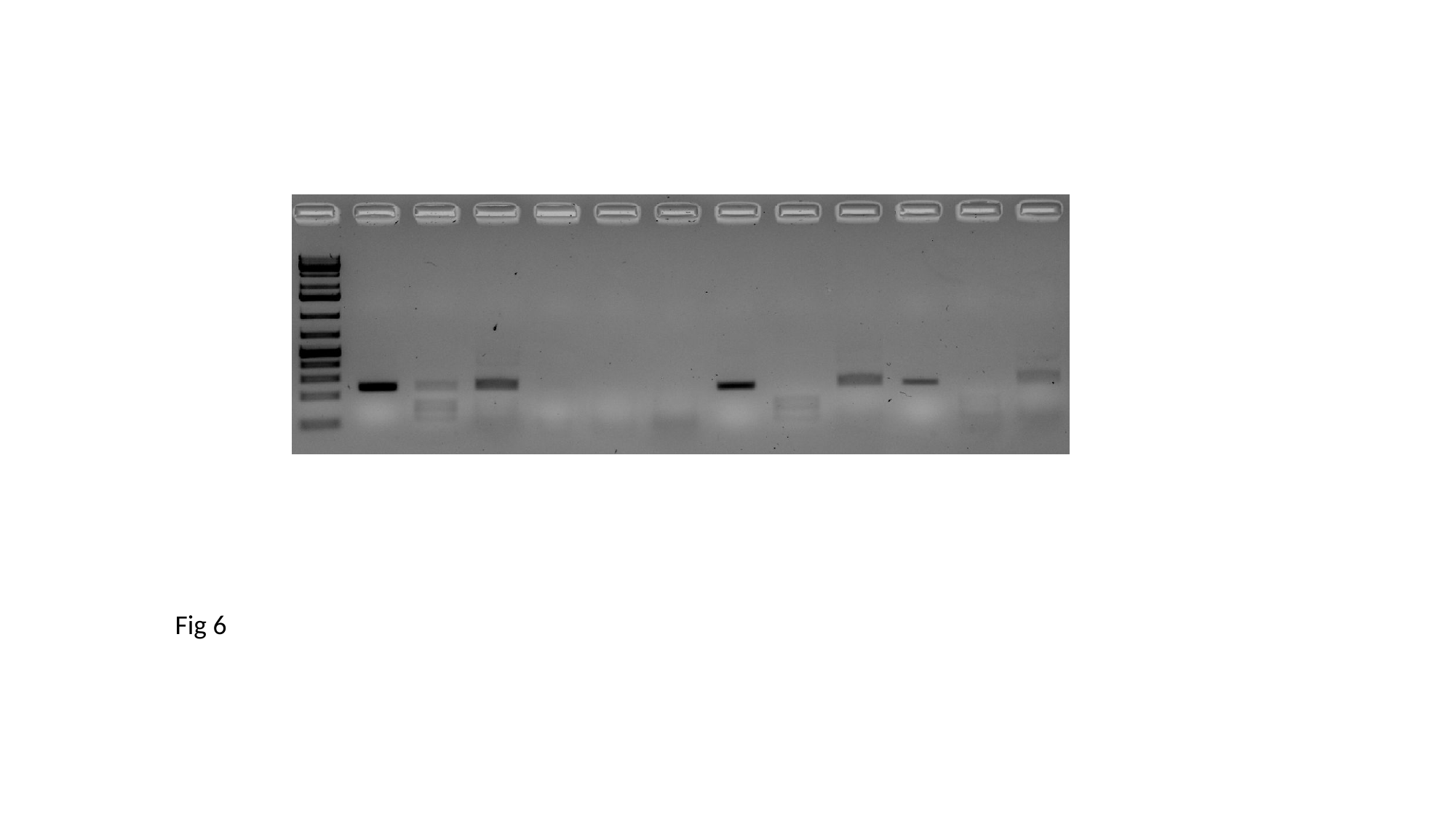

Fig 6

Supplement: Supplementary file 2 [file Presentation1.PPTX]
