# Supplementary material for: A CRISPR-based approach using dead Cas9-sgRNA to detect SARS-CoV-2
Source: Front Mol Biosci. 2023 Jun 14;10:1201347. doi: 10.3389/fmolb.2023.1201347 (PMC10300348; doi:10.3389/fmolb.2023.1201347)
Supplement: Supplementary file 3 [file Presentation2.PPTX]

## Slide 1
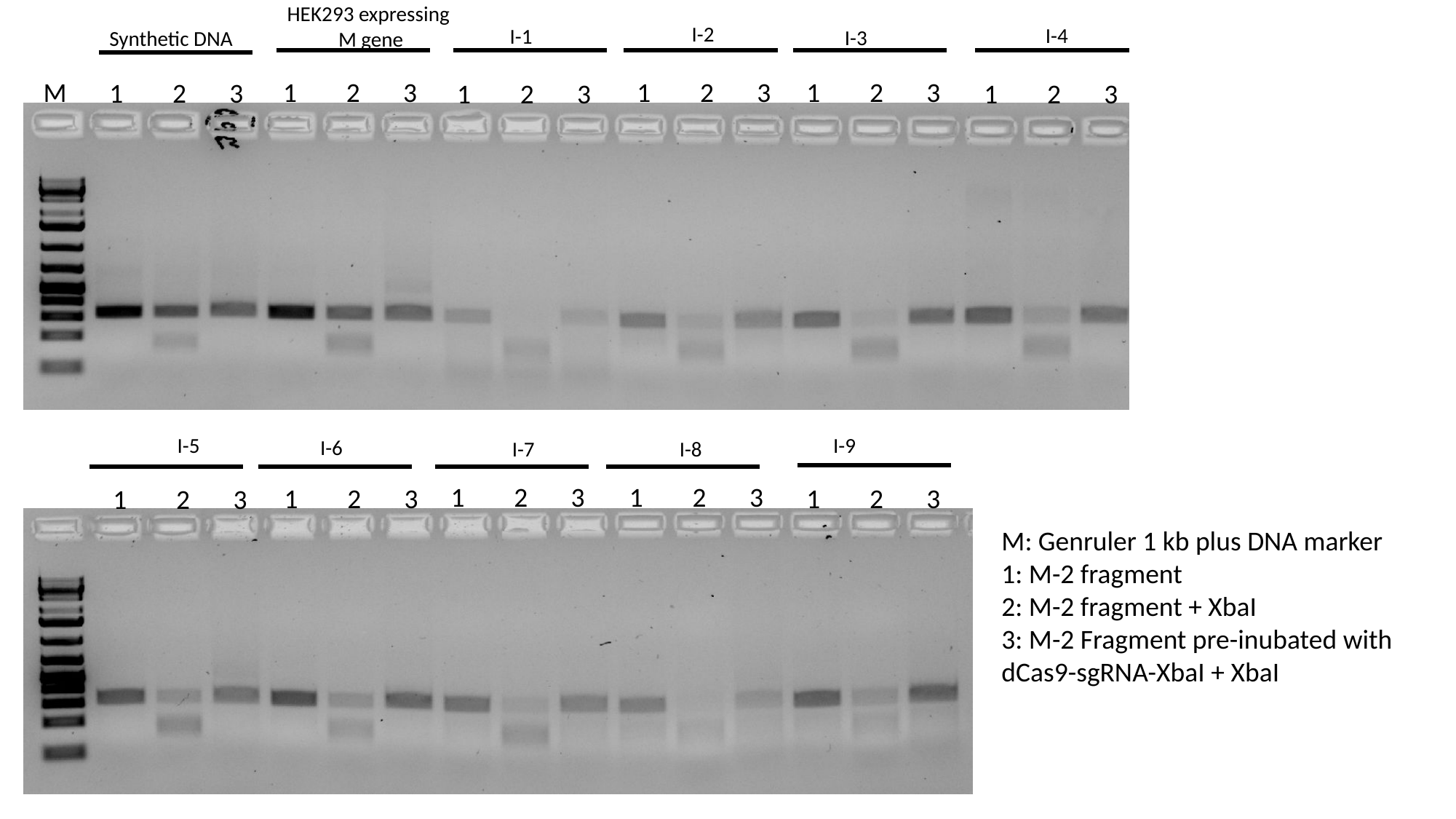

HEK293 expressing
M gene
I-2
I-4
I-1
I-3
Synthetic DNA
1 2 3
M
1 2 3
1 2 3
1 2 3
1 2 3
1 2 3
I-9
I-5
I-6
I-8
I-7
1 2 3
1 2 3
1 2 3
1 2 3
1 2 3
M: Genruler 1 kb plus DNA marker
1: M-2 fragment
2: M-2 fragment + XbaI
3: M-2 Fragment pre-inubated with dCas9-sgRNA-XbaI + XbaI
